# Association between tobacco substance usage and a missense mutation in the tumor suppressor gene P53 in the Saudi Arabian population

**DOI:** 10.1371/journal.pone.0245133

**Published:** 2021-01-22

**Authors:** Mikhlid H. Almutairi, Bader O. Almutairi, Turki M. Alrubie, Sultan N. Alharbi, Narasimha R. Parine, Abdulwahed F. Alrefaei, Ibrahim Aldeailej, Abdullah Alamri, Abdelhabib Semlali

**Affiliations:** 1 Zoology Department, College of Science, King Saud University, Riyadh, Saudi Arabia; 2 Master’s Student, Zoology Department, College of Science, King Saud University, Riyadh, Kingdom of Saudi Arabia; 3 National Center for Stem Cell Technology, King Abdulaziz City for Science and Technology, Riyadh, Saudi Arabia; 4 Genome Research Chair, Department of Biochemistry, College of Science, King Saud University, Riyadh, Kingdom of Saudi Arabia; 5 Ministry of Health, Riyadh Regional Lab Director, Riyadh, Kingdom of Saudi Arabia; 6 Groupe de Recherche en Écologie Buccale, Faculté de Médecine Dentaire, Université Laval, Québec, Canada; CNR, ITALY

## Abstract

The tumor suppressor gene TP53 and its downstream genes P21 and MDM2 play crucial roles in combating DNA damage at the G_1_/S cell cycle checkpoint. Polymorphisms in these genes can lead to the development of various diseases. This study was conducted to examine a potential association between tobacco substance usage (TSU) and single-nucleotide polymorphism (SNP) at the exon regions of the P53, P21, and MDM2 genes by comparing populations of smokers and non-smokers from Saudi Arabia. P53 rs1042522 (C/G), P21 rs1801270 (A/C), and MDM2 rs769412 (A/G) were investigated by genotyping 568 blood specimens: 283 from male/female smokers and 285 from male/female non-smokers. The results obtained from the smokers and their control non-smokers were compared according to age, sex, duration of smoking, and type of TSU. Heterozygous CG, homozygous GG, and CG+GG genotypes, as well as the G allele of rs1042522 were significantly associated with TSU in Saudi smokers compared with non-smokers. The C allele frequency of rs1801270 was also associated with TSU in smokers (OR = 1.33, p = 0.049) in comparison with non-smokers, in younger smokers (≤29 years) (OR = 1.556, p = 0.03280) in comparison with non-smokers of the same age, in smokers who had smoked cigarettes for seven years or less (OR = 1.596, p = 0.00882), and in smokers who had consumed shisha (OR = 1.608, p = 0.04104) in comparison with the controls. However, the genotypic and allelic frequencies for rs769412 did not show significant associations with TSU in Saudis. The selected SNP of P53 was strongly associated with TSU and may be linked to TSU-induced diseases in the Saudi Arabian population.

## Introduction

According to the World Health Organisation, global statistics on trends in tobacco substance usage (TSU) indicate that between the years 2000 and 2025, 1.1 billion youth under the age of 15 have smoked, are smoking, or will smoke (https://punchng.com/1-billion-people-smoke-globally-who-says/) [1]. This large number increases the death toll from TSU among males and females in developed countries by 24% [2]. This number also adds to the 10 million people worldwide who, by the year 2030, will die of diseases related to TSU as well as to the 1 billion who will die of these same causes by the end of the 21st century [3]. Several studies have reported an association between TSU and the formation of tumors in the lungs [4, 5], breasts [6, 7], mouth [8, 9], pancreas [10, 11], colon [12, 13], and kidneys [14, 15]. Associations of TSU with other diseases, including periodontal diseases, cardiovascular diseases [16], and asthma [17], have also been reported. Tobacco is known to contain several hundred different toxic substances (https://www.lung.org/quit-smoking/smoking-facts/whats-in-a-cigarette) that can directly alter genes in cells in the small airways [18] and lungs [19], as well as indirectly cause mutations in genes located in the cells in the bloodstream [20]. Two recent studies have identified the effects of TSU on 599 [21] and 290 genes [19]. This work has shed light on which genes undergo dysregulation of their expression in smokers because of their TSU [19], including cell cycle-related genes in particular [22]. In another study conducted in Ishikawa, Japan, the endometrial adenocarcinoma cells of the participants were treated with components of tobacco [23]. An association was found between TSU and the downregulation of the P16 tumor suppressor protein encoded by the CDKN2A gene that slows down cell division [24]. In addition, a reduction in expression was found to occur in the pathways of the cyclin-dependent kinase inhibitors P21 and P27, with their expression increasing inside the levels of transmission of the cyclins D1 and E. Moreover, previous studies have demonstrated the significant association between TSU and mutations in P53, which cause squamous cell carcinoma in the lungs. TSU has been shown to affect the gene expression of P53 [25] by altering its negative regulator, the MDM2 gene [26], which in turn causes MDM2 upregulation in the lungs of cancer patients who are smokers [27]. Recently, single-nucleotide polymorphisms (SNPs) have also been linked to various diseases, including celiac disease [28], rheumatoid arthritis [29], bipolar disorder [30], and asthma [31, 32], as well as lung [33], gastric [34], ovarian [35], and breast cancers [36]. Other studies have also demonstrated that TSU causes SNPs that affect gene expression in various locations in the genome [37, 38]. One study of SNPs carried out on genes involved in systematic inflammation noted that TSU causes an increase in the levels of immunosuppressive cells in the bloodstream of smokers [39]. In addition, it was found that a consistent elevation of IFN-γ was primarily caused by changes occurring at an SNP at its rs2069705 (AG/GG) position due to TSU [40]. TSU has also been reported to contribute to the development of inflammatory bowel disease in smokers [41]. Moreover, the literature has mentioned that TSU appears to play a vital role in causing genetic variations in the cell cycle regulator genes MDM2 [42], TP53 [43], and P21 [44]. In turn, they cause mutations at P53, which is involved in the P21 and MDM2 pathways, thereby leading to the development of tumors [45, 46]. For example, regarding the P53 SNP rs1042522, at codon 72 (G>C Arg to Pro), greater apoptotic activity was identified with Arg72 than with Pro72. This causes the increased expression of MDM2, P21, and caspase 3 [47]. It is also likely that the MDM2 polymorphisms rs22797944, rs937283, and rs769412 are responsible for controlling such expression and for causing disruptions in the pathways of P53 [48]. In addition, in smokers who are also alcoholics, this might contribute to increase susceptibility to the development of carcinoma of the larynx [49]. Other disruptions in the P53 pathways might result in SNP polymorphisms at the downstream P21, similar to a case in which a P21 polymorphism involving C>A modification at rs1801270 causes the progression of uterine leiomyoma [50], but decreases the risk of preeclampsia [51]. Several reports have mentioned that TSU plays an important role in the occurrence of SNP mutations [38, 52]. These include key changes in the expression of the MDM2 [53], P21 [44], and P53 genes [52, 53], which increase the likelihood of certain diseases [39–41] and cancers to occur or progress [42–44]. Against this background, this study aimed to investigate the possible association between genetic variants of P53 rs1042522, P21 rs1801270, and MDM2 rs7694412 and TSU in Saudis to better understand and identify which specific genetic markers can facilitate the diagnosis of TSU-related diseases.

## Materials and methods

### Ethical approval

Approval for this study was provided by the Research Ethics Committee of the College of Applied Medical Sciences at King Saud University in Riyadh, Saudi Arabia (Reference No. CAMS 13/3536). All smoking and non-smoking participants verbally consented and signed an agreement form providing their informed consent before taking part in our study. All of the participants were handed a privacy statement that described how their personal data would be collected, stored, and protected. The personal data of all of the participants were collected by having them fill out a questionnaire on the following demographic factors: age, allergy and disease types, smoking history, smoking habits, types of tobacco substances used, and daily levels of consumption.

### Collection of blood samples

The Saudi population sampled was made up of a total of 568 males and females, among whom 283 were smokers and 285 were non-smokers (healthy controls). We collected a 3 ml blood samples from each participant using ethylenediaminetetraacetic acid (EDTA), vacutainer vials, and blood collection tubes. All samples were collected at the Blood Donation Center of King Saud Medical City in Riyadh, Saudi Arabia, in January and February 2019. Participants who self-reported that they were suffering from an inflammatory disease or chronic respiratory failure were excluded from this study.

### Extraction of genomic DNA

Genomic DNA was extracted from 200 μl of EDTA anticoagulated peripheral blood. This extraction was performed in accordance with a standard set of procedures using a DNeasy® Blood & Tissue Kit (QIAGEN; 69504). First, 20 μl of Proteinase K solution was pipetted and deposited at the bottom of a new and clean 1.5 ml microcentrifuge tube. Second, 200 μl of blood sample was added, followed by 200 μl of BL buffer. These were mixed together by pulse vortexing for 15 s and incubated at 56°C for 10 min using an Eppendorf ThermoStat Plus. All of the tubes were briefly spun down using a mini centrifuge. Then, 200 μl volume of absolute ethanol was added to the mixture, before it was made to undergo one final 15 s spin by pulse vortexing. Subsequently, the entire batch was transferred to a QIAGEN mini-spin column fitted with a 2 ml collection tube. Upon centrifugation at 8000 rpm for 1 min, the samples were transferred into new collection tubes in which 500 μl of AW1 buffer solution had been added before another centrifugation at 8000 rpm for 1 min. Then, they were transferred to other collection tubes containing 500 μl of AW2 buffer solution, mixed by centrifugation at 8000 rpm for 1 min, and transferred into new and clean 1.5 mL microcentrifuge tubes that were each filled with 75 μl of an AE. The entire batch was incubated one final time for 1 min at room temperature, before being centrifuged at full speed (14,000 rpm) for 1 min.

### Determination of DNA concentration

The DNA concentration was quantified using a Nano-Drop 8000 spectrophotometer. Sample purity was calculated by determining the ratios of A_260_:A_280nm_ and A_260_:A_230nm_. The DNA was considered pure when its ratio was around the 2.0 mark. Once purified, the collected DNA samples were stored at −20°C for later analysis.

### SNP selection and preparation of TaqMan® SNP genotyping assay

Two selection criteria were used for the P53, P21, and MDM2 polymorphisms: (1) exon position and (2) their associations with different cancers or/and diseases in different ethnic groups, as mentioned previously. The descriptions of the selected polymorphism are presented in the S1 Table. Genomic DNA blood samples measuring 10 ng were prepared before undertaking any genotyping. Each DNA blood sample was genotyped in a reaction volume of 10 μl on a 96-well plate. Each well contained 8 μl of reaction mixture, 5 μl of a TaqMan^®^ Genotyping Master Mix (Applied Biosystems; 4371355), 40× TaqMan® Genotyping of an SNP Assay (0.25 μl), DNase-free water (2.75 μl), and 2 μl of diluted DNA that reached a final volume of 10 μl. The 96-well plate was then sealed with adhesive film and briefly centrifuged to remove any air bubbles before its content was collected. Allelic discrimination for SNPs was performed by Sequence Detection Software using the QuantStudio™ 7 Flex Real-time PCR System. Then, real-time PCR amplification was performed with the following reaction conditions: pre-denaturation for 7 min at 95°C, 40 cycles of denaturation for 30 s at 95°C, annealing for 1 min at 60°C, extension for 30 s at 72°C, and then a final extension for 5 min at 72°C.

### 3D structural analysis

A 3D structural analysis was conducted to predict any potential structural modifications and any damaging effects that might have occurred in association with the SNPs at P53, P21, and MDM2. After obtaining the protein sequences from the NCBI, data analysis was performed using one of the most widely known protein modeling, prediction, and analysis programs; the Phyre2 protein-fold recognition server [54]. This was followed by imaging of the P53, P21, and MDM2 proteins using PyMOL’s software for their 3D structures. SNPs were then introduced into their sequences to test for any potential structural modifications that might occur.

### Statistical analysis

As described in a previous paper [55], the two groups were examined using the chi-squared test to compare their allelic and genotypic frequencies. The statistical software Statistical Package for the Social Sciences version 16.0 was used to examine the statistical significance, setting a threshold of p < 0.05. A Hardy–Weinberg equilibrium test was also performed using Fisher’s two-tailed exact statistical significance test. Finally, the odd ratios (OR) at 95% confidence intervals were measured to test the strength of the connection.

## Results

### Clinical and demographic data of the studied subjects

The basic clinical data from the Saudi smokers and non-smokers who took part in this study are presented in Table 1. Overall, there were 568 participants: 283 male and female smokers, among whom 285 were male and female non-smokers. There were no significant demographic differences between the smokers and the non-smokers in terms of their age. The median ages of the participants in the two groups were almost identical (smokers 29.76±7.09 and non-smokers 29.13±8.83). Since the male-to-female ratio of the smokers was 91.5:8.5, we decided that it would be best to divide them into two groups based on their number of years of smoking. Then, we added non-smokers to act as controls to the smokers in each group. Subsequently, we tested all of our subjects to determine the effect of TSU in terms of the numbers of years smoking on altered SNP genotypes. Group 1 comprised those who had been smoking for 7 years or less (61.0%), while Group 2 comprised those who had been smoking for more than 7 years (39.0%). After calculating and obtaining their average TSU levels, we again divided them to find ascertain the association between daily TSU habits and gene mutations. Group 1 comprised smokers who consumed 12 cigarettes or less per day (51.4%), while Group 2 comprised smokers who smoked more than 12 cigarettes per day (48.6%). After obtaining data on their daily TSU habits, we again divided them into two groups, based on the types of tobacco that they consumed: cigarettes (75.5%) or shisha (24.5%). After testing, the results showed how the usage of various tobacco products influences alterations in various genes that could lead to the development of diseases in smokers.

**Table 1:** Clinical and demographic data of the study participants.

Variable	Smokers	Non-smokers
Number	283	285
Age (years), median ± SD	29.76±7.09	29.13±8.83
**Age (years)**		
≤ 29 years	152 (53.7%)	174 (61.3%)
˃ 29 years	131 (46.3%)	110 (38.7%)
**Gender**		
Males	259 (91.5%)	201 (70.5%)
Females	24 (8.5%)	84 (29.5%)
**Years of smoking**		
≤ 7 years	172 (61.0%)	---
˃ 7 years	110 (39.0%)	---
**Quantity of cigarette smoking per day**		
≤ 12 times	145 (51.4%)	---
˃ 12 times	137 (48.6%)	---
**Type of smoking**		
Cigarette	213 (75.5%)	---
Shisha	69 (24.5%)	---

### General genotypic polymorphism distributions at P53, P21, and MDM2 in smokers versus non-smokers

Three SNPs were genotyped using a TaqMan genotyping assay: rs1042522 C˃G (P72R), in the TP53 gene, rs1801270 A˃C (R31S), in the P21 gene, and rs769412 A˃G (E354E) in the MDM2 gene. The genotype and allele frequencies for the selected polymorphisms for both smokers and non-smokers (controls) are described in Table 2. A statistically significant association with smoking was found for the three SNP genotypes at the TP53 gene’s rs1042522. In the smokers, the CC, CG, and GG genotypes were present at rates of 8%, 52%, and 40%, respectively. Along with the G allele, the heterozygous CG, homozygous GG, and the combined genotypes CG+GG demonstrated significant variations when compared to CC and the C reference alleles (OR = 3.80, p < 0.00001; OR = 5.80, p < 0.00001; OR = 4.47, p < 0.00001; and OR = 2.075, p < 0.00001, respectively; Table 2). In the controls, the CC, CG, and GG genotypes were present at rates of 27%, 49%, and 24%, respectively. However, no significant correlation was found for either of the genetic variants in the smokers and non-smokers for P21’s rs1801270 and MDM2’s rs769412, with the exception of the C allele at rs1801270. For the SNP rs1801270, the AA, AC, and CC genotypes were present at rates of 5%, 27%, and 68% in the smokers versus 7%, 33%, and 60%, respectively, in the controls. Curiously, an association was found between the C allele at the variant rs1801270 and TSU, relative to the A reference allele (OR = 1.33, p = 0.049). Regarding the genotypes, the MDM2 SNP rs769412 was distributed as follows: 84% for AA, 16% for AG, and 0% for GG in the smokers, but 85% for AA, 15% for AG, and 0% for GG in the controls.

**Table 2:** General genotype distributions of TP53, P21, and MDM2 gene SNPs among smokers and non-smokers (controls).

Gene	SNP	Alleles	Controls		Smokers		OR	95% CI	X^2^	P value
			N	Percent	N	Percent				
**TP53**	rs1042522	**Total**	**285**	**100%**	**283**	**100%**				
		CC	78	27%	22	8%	Ref			
		CG	138	49%	148	52%	3.80	2.24–6.44	26.61	<0.00001*
		GG	69	24%	113	40%	5.80	3.31–10.16	41.56	<0.00001*
		CG+GG	207	73%	261	92%	4.47	2.69–7.42	37.58	<0.00001*
		C	294	52%	192	34%	Ref			
		G	276	48%	374	66%	2.075	163–2.63	36.17	<0.00001*
**P21**	rs1801270	**Total**	**274**	**100%**	**278**	**100%**				
		AA	19	7%	14	5%	Ref			
		AC	91	33%	76	27%	1.133	0.533–2.410	0.11	0.74481
		CC	164	60%	188	68%	1.556	0.756–3.201	1.46	0.22696
		AC+CC	255	93%	264	95%	1.405	0.690–2.862	0.88	0.34692
		A	129	24%	104	19%	Ref			
		C	419	76%	452	81%	1.33	1.00–1.78	3.87	0.049*
**MDM2**	rs769412	**Total**	**282**	**100%**	**282**	**100%**				
		AA	239	85%	236	84%	Ref			
		AG	42	15%	45	16%	1.085	0.687–1.714	0.12	0.72645
		GG	1	0%	1	0%	1.013	0.063–16.285	0.00	0.99289
		AG+GG	43	15%	46	16%	1.083	0.689–1.704	0.12	0.72896
		A	520	92%	517	92%	Ref			
		G	44	8%	47	8%	1.074	0.700–1.650	0.11	0.74292

*P < 0.05, SNP: Single Nucleotide Polymorphism, N = Number, Ref = Reference allele, OR: Odd Ratio.

### Correlation between age and SNPs at P53, P21, and MDM2 in smokers

To determine whether there was a correlation between the age of the smokers and the occurrence of polymorphisms at TP53, P21, and MDM2, we created two age-related cohorts of smokers and their controls. Group 1 comprised participants who were ≤29 years, while Group 2 contained those ˃29 years. Group 1 included 152 smokers and 174 non-smokers, while Group 2 included 131 smokers and 110 non-smokers (Table 1). The results for the group of smokers ≤29 years of age indicated an association between the TP53 SNP at rs1042522 for all allelic and genotypic distributions (Table 3). In these younger smokers, the G allele, and the CG, GG, and CG+GG genotypes correlated significantly with TSU (OR = 4.095, p = 0.00007 for CG; OR = 4.783, p = 0.00003 for GG; OR = 4.338, p = 0.00001 for CG+GG; and OR = 1.774, p = 0.00035 for the G allele) (Table 3). In the group of older smokers (˃29 years), the results for the same SNP regarding the CG, GG, and CG+GG genotypes and the G allele indicated significant associations with TSU (OR = 3.616, p = 0.00077 for CG; OR = 7.794, p < 0.00003 for GG; OR = 4.880, p < 0.00007 for CG+GG; and OR = 2.590, p < 0.00003 for the G allele) (Table 3).

**Table 3:** Comparison of genotype distributions of P53, P21, and MDM2 gene polymorphisms between smokers aged ≤29 years and ˃29 years and their corresponding controls.

Gene	SNP	Allele	Controls		≤ 29 years		OR	95% CI	X^2^	P value
			N	Percent	N	Percent				
**TP53**	rs1042522	**Total**	**174**	**100%**	**152**	**100%**				
		CC	44	25%	11	7%	Ref			
		CG	84	48%	86	57%	4.095	1.982–8.463	15.85	0.00007*
		GG	46	27%	55	36%	4.783	2.219–10.309	17.32	0.00003*
		CG+GG	130	75%	141	93%	4.338	2.149–8.757	18.85	0.00001*
		C	172	49%	108	36%	Ref			
		G	176	51%	196	64%	1.774	1.294–2.431	12.79	0.00035*
**P21**	rs1801270	**Total**	**165**	**100%**	**149**	**100%**				
		AA	11	7%	5	3%	Ref			
		AC	51	31%	36	24%	1.553	0.497–4.856	0.58	0.44682
		CC	103	62%	108	73%	2.307	0.775–6.868	2.36	0.12415
		AC+CC	154	93%	144	97%	2.057	0.698–6.065	1.77	0.18278
		A	73	22%	46	15%	Ref			
		C	257	78%	252	85%	1.556	1.035–2.340	4.56	0.03280*
**MDM2**	rs769412	**Total**	**173**	**100%**	**151**	**100%**				
		AA	147	85%	131	87%	Ref			
		AG	25	14%	20	13%	0.898	0.476–1.691	0.11	0.73837
		GG	1	1%	0	0%	0.374	0.015–9.258	0.89	0.34593
		AG+GG	26	15%	20	13%	0.863	0.460–1.619	0.21	0.64629
		A	319	92%	282	93%	Ref			
		G	27	8%	20	7%	0.838	0.460–1.527	0.33	0.56313
**Gene**	**SNP**	**Allele**	**Controls**		**˃ 29 years**		**OR**	**95% CI**	**X**^**2**^	**P value**
			N	Percent	N	Percent				
**TP53**	rs1042522	**Total**	**110**	**100%**	**131**	**100%**				
		CC	34	31%	11	8%	Ref			
		CG	53	48%	62	48%	3.616	1.670–7.828	11.32	0.00077*
		GG	23	21%	58	44%	7.794	3.385–17.946	25.97	<0.00003*
		CG+GG	76	69%	120	92%	4.880	2.333–10.209	19.95	<0.00007*
		C	121	55%	84	32%	Ref			
		G	99	45%	178	68%	2.590	1.787–3.754	25.74	<0.00003*
**P21**	rs1801270	**Total**	**108**	**100%**	**129**	**100%**				
		AA	8	7%	9	7%	Ref			
		AC	40	37%	40	31%	0.889	0.312–2.536	0.05	0.82566
		CC	60	56%	80	62%	1.185	0.432–3.252	0.11	0.74129
		AC+CC	100	93%	120	93%	1.067	0.397–2.867	0.02	0.89818
		A	56	26%	58	22%	Ref			
		C	160	74%	200	78%	1.207	0.791–1.840	0.76	0.38208
**MDM2**	rs769412	**Total**	**108**	**100%**	**131**	**100%**				
		AA	91	84%	105	80%	Ref			
		AG	17	16%	25	19%	1.275	0.648–2.508	0.49	0.48196
		GG	0	0%	1	1%	2.602	0.105–64.655	0.86	0.35294
		AG+GG	17	16%	26	20%	1.325	0.676–2.597	0.68	0.41076
		A	199	92%	235	90%	Ref			
		G	17	8%	27	10%	1.345	0.712–2.539	0.84	0.35942

*P < 0.05, SNP: Single Nucleotide Polymorphism, N = Number, Ref = Reference allele, OR: Odd Ratio.

Among the younger smokers (≤29 years), the AA, AC, and CC genotypes for the P21 SNP rs1801270 were found at rates of 3%, 24%, and 73% in the smokers, versus 7%, 31%, and 62% in the controls, respectively. When the younger smokers were compared to their non-smoking controls, the heterozygous AC and homozygous CC frequencies demonstrated no significant association with TSU, with the exception of the C allele frequency, which was strongly associated with TSU compared with the A reference allele (OR = 1.556, p = 0.03280). In the older smokers (˃29 years), who were also compared with their controls, no significant differences were found. The genotypes AA, AC, and CC for the SNP rs1801270 were found at rates of 7%, 31%, and 62% in the older smokers versus 7%, 37%, and 56% in the older non-smokers. When the C allele frequency in the older smokers was compared to that of the A reference allele, the association of the former was not significant (Table 3). For the MDM2 SNP rs769412, the association between TSU and age was also insignificant in both the younger and the older smokers, as shown in Table 3. In the younger group, the genotypes for this SNP were 87% for AA, 13% for AG, and 0% for GG in the smokers versus 85% for AA, 14% for AG, and 1% for GG in the non-smokers. Allele frequencies were 93% for A and 7% for G in the smokers versus 92% for A and 8% for G in the non-smokers (Table 3). Meanwhile, in the older smokers, the genotype frequencies were 80% for AA, 19% for AG, and 1% for GG versus 84% for AA, 16% for AG, and 0% for GG in the non-smokers. The allele frequencies were 90% for A and 10% for G in the smokers versus 92% for A and 8% for G in the non-smokers (Table 3).

### Correlation between sex and SNPs in P53, P21, and MDM2 in smokers

To test the existence of associations among TSU, sex, and the SNPs in P53, P21, and MDM2, we divided the participants into two groups of males and females, including both smokers and their non-smoking controls. Group 1 comprised 259 male smokers and 201 male non-smokers, while Group 2 comprised 24 female smokers and 84 female non-smokers. The results indicated that the polymorphism rs1042522 in the P53 gene was associated with both male and female smokers rather than with non-smokers. As presented in Table 4, the CG, GG, and CG+GG genotypes, along with the G allele, presented strong associations with TSU in the male smokers versus the male non-smoking controls (OR = 3.510, p < 0.00005; OR = 5.213, p < 0.00002; OR = 4.058, p < 0.00007; and OR = 1.982, p < 0.00003, respectively). Similar significant results were obtained for the females (Table 4), although the cohort of female smokers was markedly smaller than that of their controls. Specifically, the CG, GG, and CG+GG genotypes and the G allele were strongly associated with TSU in the female smokers versus the non-smokers (OR = 11.304, p = 0.02626; OR = 32.378, p = 0.00042; OR = 18.724, p = 0.00386; and OR = 4.438, p = 0.00009, respectively). However, our results failed to prove the existence of any significant relationship between the polymorphism rs1801270 located in the P21 gene and TSU in either sex. In males, the genotypes AA, AC, and CC for this polymorphism were present at rates of 5%, 27%, and 68% in the smokers versus 7%, 31%, and 62% in the non-smokers, respectively (Table 4). In females, the same SNP genotype rates were 0%, 39%, and 61% in the smokers, versus 7%, 38%, and 55% in their controls, respectively. Moreover, in both sexes, the results obtained for the MDM2 SNP rs769412 indicated an insignificant association between the genotypic frequencies and TSU. The genotype frequencies in male and female smokers were 84% and 83% for the AA reference genotype, 16% and 17% for the heterozygous AG, and 0% for both double mutants GG. By contrast, in both males and females in the cohorts of controls for this variant, the rates were 83% and 89% for the AA reference genotype, 17% and 10% for the heterozygous AG, and 0% and 1% for the double mutant GG.

**Table 4:** Genotype and allele frequencies of SNPs in P53, P21, and MDM2 genes in male and female smokers and controls.

Gene	SNP	Allele	Controls		Male		OR	95% CI	X^2^	P value
			N	Percent	N	Percent				
**TP53**	rs1042522	**Total**	**201**	**100%**	**259**	**100%**				
		CC	55	27%	22	8%	Ref			
		CG	99	49%	139	54%	3.510	2.010–6.130	20.72	<0.00005*
		GG	47	24%	98	38%	5.213	2.848–9.541	30.82	<0.00002*
		CG+GG	146	73%	237	92%	4.058	2.375–6.934	28.91	<0.00007*
		C	209	52%	183	35%	Ref			
		G	193	48%	335	65%	1.982	1.519–2.586	25.70	<0.00003*
**P21**	rs1801270	**Total**	**194**	**100%**	**255**	**100%**				
		AA	13	7%	14	5%	Ref			
		AC	61	31%	67	27%	1.020	0.444–2.341	0.00	0.96291
		CC	120	62%	174	68%	1.346	0.611–2.966	0.55	0.45921
		AC+CC	181	93%	241	95%	1.236	0.567–2.695	0.29	0.59292
		A	87	22%	95	19%	Ref			
		C	301	78%	415	81%	1.263	0.911–1.750	1.96	0.16107
**MDM2**	rs769412	**Total**	**198**	**100%**	**258**	**100%**				
		AA	164	83%	216	84%	Ref			
		AG	34	17%	41	16%	0.916	0.557–1.506	0.12	0.72838
		GG	0	0%	1	0%	2.279	0.092–56.315	0.76	0.38403
		AG+GG	34	17%	42	16%	0.938	0.571–1.540	0.06	0.79987
		A	362	91%	473	92%	Ref			
		G	34	9%	43	8%	0.968	0.605–1.549	0.02	0.89186
**Gene**	**SNP**	**Allele**	**Controls**		**Female**		**OR**	**95% CI**	**X**^**2**^	**P value**
			N	Percent	N	Percent				
**TP53**	rs1042522	**Total**	**84**	**100%**	**24**	**100%**				
		CC	23	27%	0	0%	Ref			
		CG	39	47%	9	37%	11.304	0.629–203.248	4.94	0.02626*
		GG	22	26%	15	63%	32.378	1.827–573.843	12.43	0.00042*
		CG+GG	61	73%	24	100%	18.724	1.094–320.471	8.35	0.00386*
		C	85	51%	9	19%	Ref			
		G	83	49%	39	81%	4.438	2.023–9.733	15.40	0.00009*
**P21**	rs1801270	**Total**	**80**	**100%**	**23**	**100%**				
		AA	6	7%	0	0%	Ref			
		AC	30	38%	9	39%	4.049	0.208–78.705	1.73	0.18831
		CC	44	55%	14	61%	4.236	0.225–79.876	1.85	0.17334
		AC+CC	74	93%	23	100%	4.101	0.223–75.545	1.83	0.17593
		A	42	26%	9	20%	Ref			
		C	118	74%	37	80%	1.463	0.652–3.286	0.86	0.35456
**MDM2**	rs769412	**Total**	**84**	**100%**	**24**	**100%**				
		AA	75	89%	20	83%	Ref			
		AG	8	10%	4	17%	1.875	0.512–6.864	0.92	0.33655
		GG	1	1%	0	0%	1.228	0.048–31.272	0.27	0.60608
		AG+GG	9	11%	4	17%	1.667	0.465–5.976	0.62	0.42933
		A	158	94%	44	92%	Ref			
		G	10	6%	4	8%	1.436	0.430–4.801	0.35	0.52496

*P < 0.05, SNP: Single Nucleotide Polymorphism, N = Number, Ref = Reference allele, OR: Odd Ratio.

### Correlation between daily TSU and SNPs in P53, P21, and MDM2 genes in smokers

According to the daily TSU levels, we divided the 282 smokers into two groups: moderate smokers who smoked ≤12 times per day (n = 145) and heavy smokers who smoked ˃12 times per day (n = 137). This way, we were able to discover how their daily TSU levels were associated with changes in the P53, P21, and MDM2 genes. The results of the tests that we conducted indicated the presence of a significant association between smoking moderately and heavily and the mutation of the P53 SNP rs1042522. In moderate smokers, CG, GG, and CG+GG genotypes and the G allele of the P53 SNP were significantly affected by TSU compared with the non‐smokers (OR = 3.068, p = 0.00035; OR = 4.441, p < 0.00005; OR = 3.526, p = 0.00002; and OR = 1.905, p = 0.00001, respectively; Table 5). In the heavy smokers, there were also significant effects of TSU compared with the non-smokers (CG: OR = 5.087, p = 0.00001; GG: OR = 8.054, p < 0.00002; CG+GG: OR = 6.076, p < 0.00002; and G: OR = 2.251, p < 0.00001; Table 5). By contrast, analysis of the P21 SNP rs1801270 indicated that it had no association with TSU in either the moderate or the heavy smokers, even in comparison with the controls. The genotype frequencies were distributed as follows: 5% for AA, 28% for AC, and 67% for CC in the moderate smokers; 6% for AA, 26% for AC, and 68% for CC in the heavy smokers; and 7% for AA, 33% for AC, and 60% for CC in the controls. The allelic distributions for the SNP were as follows: 18% for the A allele and 82% for the C allele in the moderate smokers; 19% for the A allele and 81% for the C allele in the heavy smokers; and 24% for the A allele and 67% for the C allele in the controls. Next, we compared the rates of the MDM2 SNP rs769412 in the moderate and heavy smokers relative to their controls. No association was found between the rates of the MDM2 SNP rs769412 and the TSU levels in either of the two categories of smokers. The genotype distributions were as follows: 86% for AA, 14% for AG, and 0% for GG in the moderate smokers; 81% for AA, 18% for AG, and 1% for GG in the heavy smokers (Table 5); and 85% for AA, 15% for AG, and 0% for GG in the controls. In terms of allelic variation, the frequencies were 93% for the A allele and 90% for the G allele in the moderate smokers; 7% for the A allele and 10% for the G allele in the heavy smokers; and 92% for the A allele and 8% for the G allele in the controls.

**Table 5:** Comparison of genotype frequencies of P53, P21, and MDM2 gene polymorphisms in smokers smoking ≤12 times/day and ˃12 times/day.

Gene	SNP	Allele	Controls		≤12 times/day		OR	95% CI	X^2^	P value
			N	Percent	N	Percent				
**TP53**	rs1042522	**Total**	**285**	**100%**	**145**	**100%**				
		CC	78	27%	14	10%	Ref			
		CG	138	49%	76	52%	3.068	1.628–5.785	12.77	0.00035*
		GG	69	24%	55	38%	4.441	2.272–8.680	20.63	<0.00005*
		CG+GG	207	73%	131	90%	3.526	1.917–6.486	17.93	0.00002*
		C	294	52%	104	36%	Ref			
		G	276	48%	186	64%	1.905	1.424–2.548	19.10	0.00001*
**P21**	rs1801270	**Total**	**274**	**100%**	**142**	**100%**				
		AA	19	7%	6	5%	Ref			
		AC	91	33%	39	28%	1.357	0.504–3.658	0.37	0.54499
		CC	164	60%	95	67%	1.834	0.708–4.753	1.60	0.20597
		AC+CC	255	93%	134	95%	1.664	0.649–4.266	1.15	0.28448
		A	129	24%	51	18%	Ref			
		C	419	76%	229	82%	1.382	0.963–1.985	3.09	0.07879
**MDM2**	rs769412	**Total**	**282**	**100%**	**143**	**100%**				
		AA	239	85%	123	86%	Ref			
		AG	42	15%	20	14%	0.925	0.521–1.645	0.07	0.79127
		GG	1	0%	0	0%	0.646	0.026–15.985	0.51	0.47345
		AG+GG	43	15%	20	14%	0.904	0.509–1.603	0.12	0.72933
		A	520	92%	266	93%	Ref			
		G	44	8%	20	7%	0.889	0.513–1.538	0.18	0.67299
**Gene**	**SNP**	**Allele**	**Controls**		**˃12 times/day**		**OR**	**95% CI**	**X**^**2**^	**P value**
			N	Percent	N	Percent				
**TP53**	rs1042522	**Total**	**285**	**100%**	**137**	**100%**				
		CC	78	27%	8	6%	Ref			
		CG	138	49%	72	52%	5.087	2.329–11.113	19.31	0.00001*
		GG	69	24%	57	42%	8.054	3.591–18.065	31.05	<0.00002*
		CG+GG	207	73%	129	94%	6.076	2.841–12.994	26.43	<0.00002*
		C	294	52%	88	32%	Ref			
		G	276	48%	186	68%	2.251	1.664–3.046	28.29	<0.00001*
**P21**	rs1801270	**Total**	**274**	**100%**	**137**	**100%**				
		AA	19	7%	8	6%	Ref			
		AC	91	33%	36	26%	0.940	0.378–2.338	0.02	0.89338
		CC	164	60%	93	68%	1.347	0.567–3.197	0.46	0.49836
		AC+CC	255	93%	129	94%	1.201	0.512–2.819	0.18	0.67276
		A	129	24%	52	19%	Ref			
		C	419	76%	222	81%	1.314	0.916–1.885	2.21	0.13676
**MDM2**	rs769412	**Total**	**282**	**100%**	**138**	**100%**				
		AA	239	85%	112	81%	Ref			
		AG	42	15%	25	18%	1.270	0.738–2.188	0.75	0.38779
		GG	1	0%	1	1%	2.134	0.132–34.427	0.30	0.58447
		AG+GG	43	15%	26	19%	1.290	0.755–2.206	0.87	0.35070
		A	520	92%	249	90%	Ref			
		G	44	8%	27	10%	1.281	0.775–2.118	0.94	0.33227

*P < 0.05, SNP: Single Nucleotide Polymorphism, N = Number, Ref = Reference allele, OR: Odd Ratio.

### Association between number of years smoking and frequencies of SNPs at P53, P21, and MDM2 in smokers

To further investigate any associations between TSU in terms of number of years smoking and changes occurring in the P53, P21, and MDM2 genes, we divided our smokers into two groups based on the number of years that they had smoked. Group 1 comprised 172 short-term smokers who had smoked for ≤7 years, while Group 2 comprised 110 long-term smokers who had smoked for ˃7 years. Table 6 describes of the statistical analysis and the differences in SNP genotypes between the short-term and long-term smokers, and their corresponding controls. There were significant associations in the frequencies of the genotypes CG, GG, and CG+GG and the G allele of the P53 SNP rs1042522 in both the short-term and long-term smokers versus their controls. In the short-term smokers, the OR ratios and p values were: i) OR = 2.959 and p = 0.00021; ii) OR = 4.389 and p < 0.00001; iii) OR = 3.436 and p < 0.00008; and iv) OR = 1.914 and p < 0.00003, respectively. In the long-term smokers they were: i) OR = 6.557 and p = 0.00002; ii) OR = 10.626 and p < 0.00004; iii) OR = 7.913 and p < 0.00006; and, iv) OR = 2.381 and p < 0.00001, respectively (Table 6). When both the short- and long-term smokers were compared with the controls, the frequencies of their genotypes at the P21 SNP rs1801270 demonstrated no significant association with TSU. The genotype distributions were as follows: 4% for AA, 25% for AC, and 71% for CC in the short-term smokers; 7% for AA, 31% for AC, and 62% for CC in the long-term smokers; and 7% for AA, 33% for AC, and 60% for CC in the controls. In addition, the frequency of the C allele presented a significant association with the short-term smokers in comparison to their controls (OR = 1.596, p = 0.00882), but failed to present any association in the long-term smokers (OR = 1.059, p = 0.76203). Furthermore, in both groups, the MDM2 SNP rs769412 presented no significant association with TSU over a number of years. The genotypes AA, AG, and GG were present at rates of 86%, 14%, and 0% in the short-term smokers; 80%, 19%, and 1% in the long-term smokers (Table 6); and 85%, 15%, and 0% in the controls, respectively. For the wild‐type A allele, it was present at a rate of 93% in the short-term smokers, 89% in the long-term smokers, and 92% in the non-smokers. For the mutant G allele, it was present at 7% in the short-term smokers, 11% in the long-term smokers, and 8% in the non-smokers (Table 6).

**Table 6:** Genotype frequencies of P53, P21, and MDM2 gene SNPs in smokers smoking for ≤7 years and ˃7 years and in their controls.

Gene	SNP	Allele	Controls		≤ 7 Years		OR	95% CI	X^2^	P value
			N	Percent	N	Percent				
**TP53**	rs1042522	**Total**	**285**	**100%**	**172**	**100%**				
		CC	78	27%	17	10%	Ref			
		CG	138	49%	89	52%	2.959	1.643–5.330	13.78	0.00021*
		GG	69	24%	66	38%	4.389	2.352–8.189	23.22	<0.00001*
		CG+GG	207	73%	155	90%	3.436	1.954–6.041	19.91	<0.00008*
		C	294	52%	123	36%	Ref			
		G	276	48%	221	64%	1.914	1.454–2.520	21.65	<0.00003*
**P21**	rs1801270	**Total**	**274**	**100%**	**167**	**100%**				
		AA	19	7%	6	4%	Ref			
		AC	91	33%	42	25%	1.462	0.544–3.925	0.57	0.44965
		CC	164	60%	119	71%	2.298	0.891–5.928	3.10	0.07813
		AC+CC	255	93%	161	96%	1.999	0.782–5.112	2.17	0.14105
		A	129	24%	54	16%	Ref			
		C	419	76%	280	84%	1.596	1.123–2.270	6.86	0.00882*
**MDM2**	rs769412	**Total**	**282**	**100%**	**168**	**100%**				
		AA	239	85%	145	86%	Ref			
		AG	42	15%	23	14%	0.903	0.521–1.562	0.13	0.71432
		GG	1	0%	0	0%	0.549	0.022–13.558	0.61	0.43640
		AG+GG	43	15%	23	14%	0.882	0.510–1.523	0.20	0.65141
		A	520	92%	313	93%	Ref			
		G	44	8%	23	7%	0.868	0.515–1.466	0.28	0.59710
**Gene**	**SNP**	**Allele**	**Controls**		**˃ 7 years**		**OR**	**95% CI**	**X**^**2**^	**P value**
			N	Percent	N	Percent				
**TP53**	rs1042522	**Total**	**285**	**100%**	**110**	**100%**				
		CC	78	27%	5	4%	Ref			
		CG	138	49%	58	53%	6.557	2.524–17.035	18.53	0.00002*
		GG	69	24%	47	43%	10.626	3.999–28.234	29.82	<0.00004*
		CG+GG	207	73%	105	96%	7.913	3.109–20.140	24.91	<0.00006*
		C	294	52%	68	31%	Ref			
		G	276	48%	152	69%	2.381	1.712–3.311	27.32	<0.00001*
**P21**	rs1801270	**Total**	**274**	**100%**	**111**	**100%**				
		AA	19	7%	8	7%	Ref			
		AC	91	33%	34	31%	0.887	0.355–2.216	0.07	0.79793
		CC	164	60%	69	62%	0.999	0.418–2.391	0.00	0.99863
		AC+CC	255	93%	103	93%	0.959	0.407–2.261	0.01	0.92433
		A	129	24%	50	23%	Ref			
		C	419	76%	172	77%	1.059	0.730–1.536	0.09	0.76203
**MDM2**	rs769412	**Total**	**282**	**100%**	**113**	**100%**				
		AA	239	85%	90	80%	Ref			
		AG	42	15%	22	19%	1.391	0.787–2.460	1.30	0.25503
		GG	1	0%	1	1%	2.656	0.164–42.907	0.51	0.47455
		AG+GG	43	15%	23	20%	1.420	0.810–2.490	1.51	0.21897
		A	520	92%	202	89%	Ref			
		G	44	8%	24	11%	1.404	0.832–2.369	1.63	0.20187

*P < 0.05, SNP: Single Nucleotide Polymorphism, N = Number, Ref = Reference allele, OR: Odd Ratio.

### Comparative study of the effects of tobacco and shisha smoking on P53, P21, and MDM2 polymorphisms

To evaluate the risk associated with the type of TSU smoked, we regrouped the smokers into two categories. Group 1 was made up of 213 individuals who smoked cigarettes and Group 2 of 69 individuals who smoked shisha. For P53 SNP rs1042522, the genotype distributions and allele frequencies were significantly associated with TSU in both groups of smokers versus their controls. In the cigarette smokers, the heterozygous CG demonstrated an approximately five-fold increased association (OR = 4.783; CI = 2.526–9.055; p < 0.00003) for the type of substance smoked (tobacco) and the homozygous GG genotype manifested an eight-fold association (OR = 7.826; CI = 4.022–15.228; p < 0.00006) (Table 7). In addition, grouping the two genotypes CG+GG together yielded a 5.797-fold increase in association (OR = 5.797; CI = 3.123–10.760; p < 0.00001), while the G phenotype showed more than a two-fold association in comparison with the C phenotype (OR = 2.271; CI = 1.748–2.951; p < 0.00005). In the shisha smokers, for the same SNP, the CG genotype showed more than a two-fold increase in correlation with the type of substance smoked (OR = 2.324; CI = 1.066–5.067; p = 0.03050), while the GG genotype presented an approximately three-fold increase in association compared with the reference CC (OR = 2.889; CI = 1.252–6.665; p = 0.01054). For the combination of the two genotypes CG+GG, a 2.512-fold increase in association with shisha smoking was noted (OR = 2.512; CI = 1.190–5.304; p = 0.01314), in contrast to the G allele phenotype, which presented a slightly lower drop in its results (OR = 1.608; CI = 1.101–2.347; p = 0.01345). Moreover, the genotype frequency of the P21 SNP rs1801270 demonstrated no significant type of TSU association in either group of smokers when they were compared to their controls. The rates of AA, AC, and CC genotypes in the cigarette and shisha smokers were as follows: 6% and 3% for AA, 28% and 25% for AC and 66% and 72% for CC, respectively. In the non-smokers, the results were 7% for AA, 33% for AC, and 60% for CC. Although the frequency of the C allele did not demonstrate any significant association with the type of TSU in the group of smokers who smoked cigarettes (OR = 1.262, p = 0.14373), an association was found in the smokers of shisha versus their controls (OR = 1.608, p = 0.04104). Regarding the results of the MDM2 SNP rs769412, no significant association was found between the types of TSU in either group of smokers when compared with their controls. The genotype distribution for AA, AG, and GG in the cigarette smokers was found to be 84%, 16%, and 0% versus 82%, 18%, and 0% in the shisha smokers (Table 7), while it was 85%, 15%, and 0% in the controls, respectively. Finally, the frequency of the G allele presented no significant association in either group of smokers when compared with their controls, as shown in Table 7.

**Table 7:** Distribution of genotype frequencies of selected SNPs in P53, P21, and MDM2 genes in smokers smoking cigarettes and shisha.

Gene	SNP	Allele	Controls		Cigarettes		OR	95% CI	X^2^	P value
			N	Percent	N	Percent				
**TP53**	rs1042522	**Total**	**285**	**100%**	**213**	**100%**				
		CC	78	27%	13	6%	Ref			
		CG	138	49%	110	52%	4.783	2.526–9.055	26.04	<0.00003*
		GG	69	24%	90	42%	7.826	4.022–15.228	42.78	<0.00006*
		CG+GG	207	73%	200	94%	5.797	3.123–10.760	36.91	<0.00001*
		C	294	52%	136	32%	Ref			
		G	276	48%	290	68%	2.271	1.748–2.951	38.39	<0.00005*
**P21**	rs1801270	**Total**	**274**	**100%**	**209**	**100%**				
		AA	19	7%	12	6%	Ref			
		AC	91	33%	58	28%	1.009	0.456–2.233	0.00	0.98205
		CC	164	60%	139	66%	1.342	0.629–2.862	0.58	0.44520
		AC+CC	255	93%	197	94%	1.223	0.580–2.580	0.28	0.59618
		A	129	24%	82	20%	Ref			
		C	419	76%	336	80%	1.262	0.924–1.723	2.14	0.14373
**MDM2**	rs769412	**Total**	**282**	**100%**	**213**	**100%**				
		AA	239	85%	179	84%	Ref			
		AG	42	15%	33	16%	1.049	0.639–1.722	0.04	0.84963
		GG	1	0%	1	0%	1.335	0.083–21.491	0.04	0.83788
		AG+GG	43	15%	34	16%	1.056	0.647–1.723	0.05	0.82815
		A	520	92%	391	92%	Ref			
		G	44	8%	35	8%	1.058	0.666–1.681	0.06	0.81163
**Gene**	**SNP**	**Allele**	**Controls**		**Shisha**		**OR**	**95% CI**	**X**^**2**^	**P value**
			N	Percent	N	Percent				
**TP53**	rs1042522	**Total**	**285**	**100%**	**69**	**100%**				
		CC	78	27%	9	13%	Ref			
		CG	138	49%	37	54%	2.324	1.066–5.067	4.68	0.03050*
		GG	69	24%	23	33%	2.889	1.252–6.665	6.54	0.01054*
		CG+GG	207	73%	60	87%	2.512	1.190–5.304	6.15	0.01314*
		C	294	52%	55	40%	Ref			
		G	276	48%	83	60%	1.608	1.101–2.347	6.11	0.01345*
**P21**	rs1801270	**Total**	**274**	**100%**	**68**	**100%**				
		AA	19	7%	2	3%	Ref			
		AC	91	33%	17	25%	1.775	0.378–8.331	0.54	0.46200
		CC	164	60%	49	72%	2.838	0.639–12.614	2.04	0.15340
		AC+CC	255	93%	66	97%	2.459	0.559–10.823	1.51	0.21956
		A	129	24%	21	15%	Ref			
		C	419	76%	115	85%	1.686	1.017–2.794	4.17	0.04104*
**MDM2**	rs769412	**Total**	**282**	**100%**	**68**	**100%**				
		AA	239	85%	56	82%	Ref			
		AG	42	15%	12	18%	1.219	0.603–2.467	0.31	0.58059
		GG	1	0%	0	0%	1.413	0.057–35.142	0.23	0.62848
		AG+GG	43	15%	12	18%	1.191	0.590–2.406	0.24	0.62563
		A	520	92%	124	91%	Ref			
		G	44	8%	12	9%	1.144	0.587–2.230	0.16	0.69330

*P < 0.05, SNP: Single Nucleotide Polymorphism, N = Number, Ref = Reference allele, OR: Odd Ratio.

### Structural and functional analyses of P53, P21, and MDM2 polymorphisms

**Fig 1:**
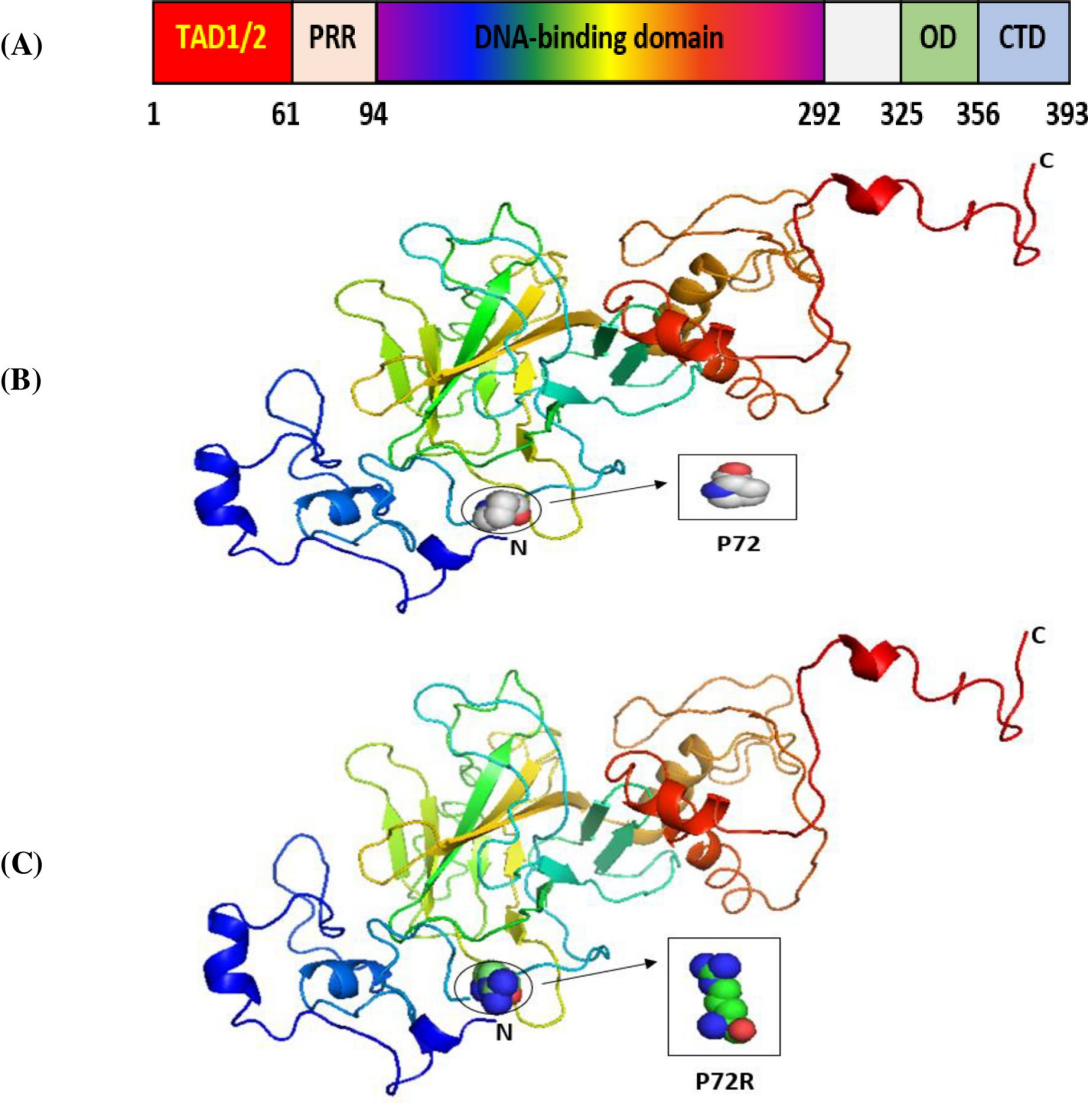
The 3D structures of normal and mutated P53 proteins. Panel (A) shows the major domain structure of a P53 protein. Panel (B) shows the 3D structure of the wild-type P53 protein (P72). Panel (C) shows the 3D structure of a P53 protein with an SNP (P72R).

The SNPs of the P53, P21, and MDM2 genes that we selected were located in exonic regions. We thus examined them for their potential effects on the P53, P21, and MDM2 proteins by using the Phyre2 server and PyMOL software [54]. The overall structure of the domains of the P53 protein is presented in Fig 1A. Its main domain is the transactivation domain (TAD) in the N-terminal region. This is divided into two basic subdomains, namely, TAD1 and TAD2, with the latter being a proline-rich region (PRR). This is followed by its core region, the DNA-binding domain (DBD), and its linker region, which connects its core domain with its tetramerization domain (OD). Its last domain, the C-terminal domain, is located in the C-terminal region [56].

**Fig 2:**
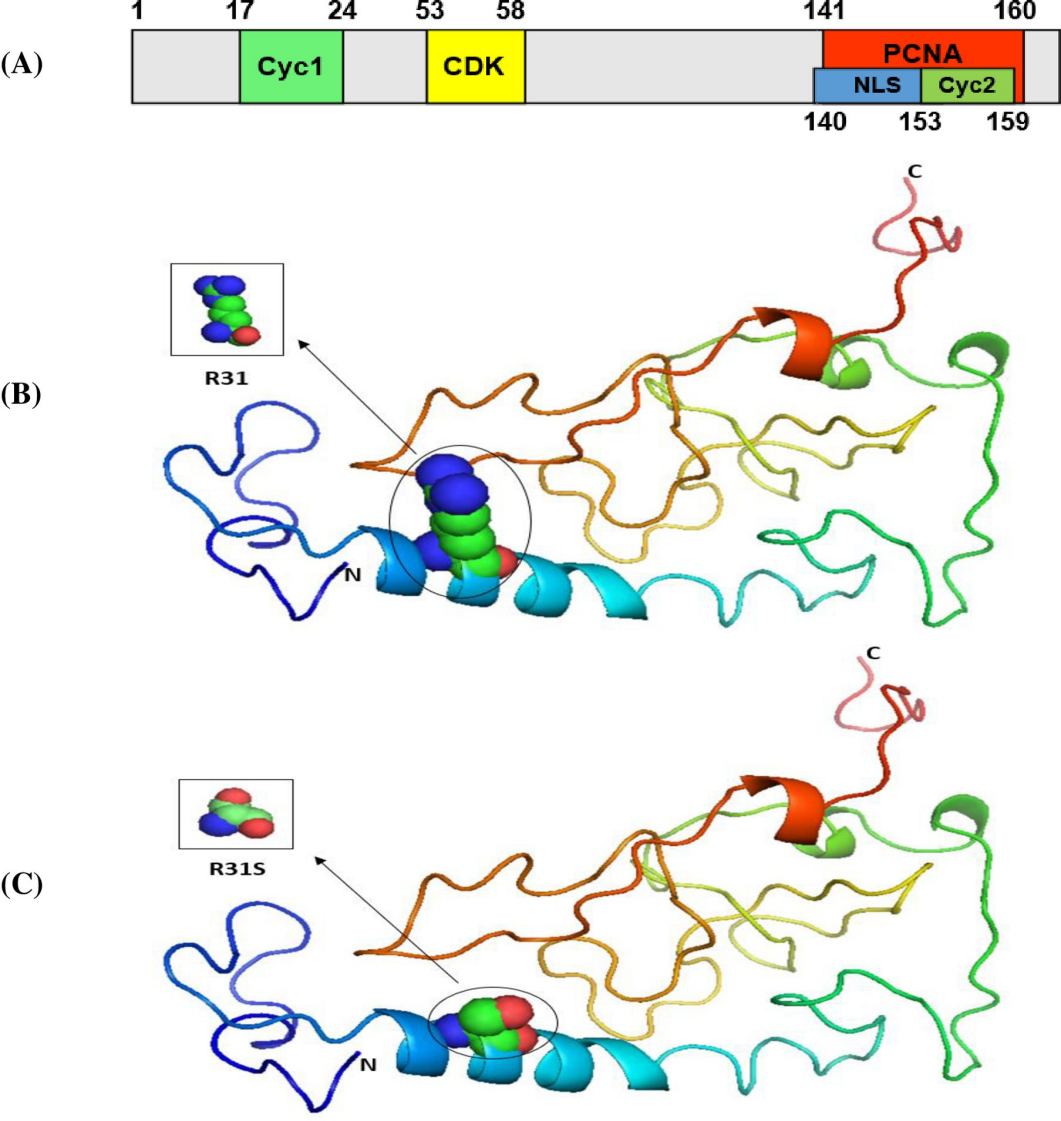
The 3D structures of normal and mutated P21 proteins. Panel (A) shows the major domain structure of a P21 protein. Panel (B) shows the 3D structure of a normal P21 protein (R31). Panel (C) shows the 3D structure of a P21 protein with an SNP (R31S).

Our results indicated that the wild-type structure of the P53 protein includes a proline amino acid, which is located at position 72 in the PRR. It displays approximately 61 to 94 residues of the P53 protein (Fig 1B). This is contrary to what we discovered that occurs for the SNP P72R at rs1042522, whereby structure and functioning are affected by the P53 protein, especially at the PRR level, and where proline is substituted by an arginine that is normally located at number 72 on P53 (Fig 1C). In other words, this polymorphism becomes damaging by association with TSU. For the P21 protein, the main domain is presented in Fig 2A. Within it are two subdomains, namely, Cyc1 and Cyc2, located in the N- and C-terminal regions, respectively. P21 contains other domains, such as CDK, which is also located in the C-terminal region, and PCNA, which can be found in the NLS region [57].

**Fig 3:**
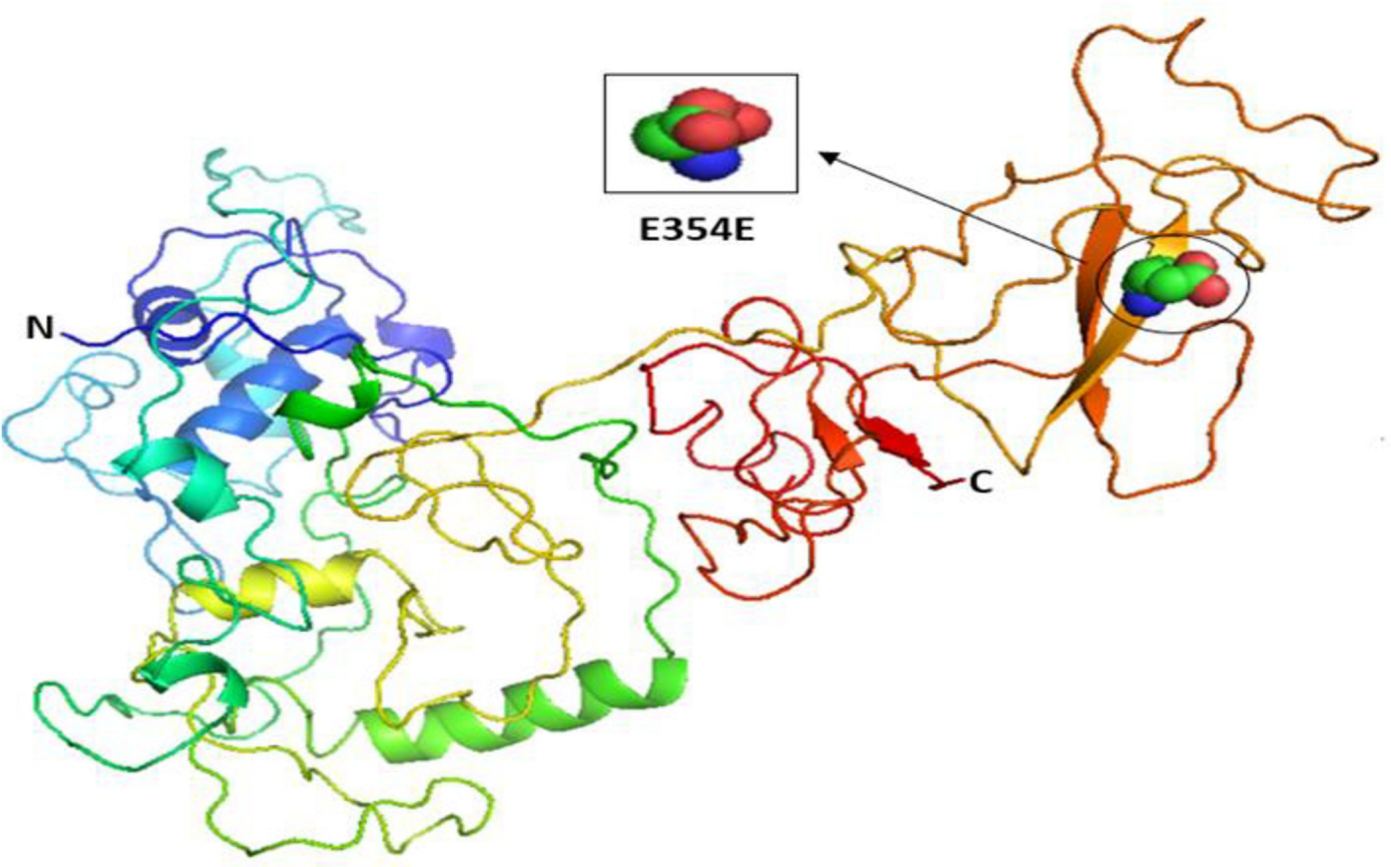
The 3D structures of normal and mutated MDM2 proteins.

Our results indicated that the wild-type structure of P21 usually has an arginine amino acid located at position 31 (Fig 2B), but R31S at rs1801270 replaces its residue with a serine amino acid (Fig 2C). In other words, R31S, a coding-region non-synonymous SNP, transforms P21’s amino acid sequence without affecting its functional domains. Therefore, we found no association between it and TSU in the sample Saudi population included in this study. Based on the results obtained using Phyre2 and PyMOL, the structure of the MDM2 protein does not change with the E354E SNP rs769412, given its synonymous status. This is because a coding SNP cannot change the sequence of MDM2. Similar amino acid (glutamic acid) levels to those found at MDM2's number 354 were found to be present at its location (Fig 3). As such, the SNP E354E is not associated with TSU in Saudi smokers.

### Population results from Saudi Arabia compared with those from other countries

Comparative studies on the genotyping results for the selected SNPs in non-smokers were conducted on samples collected from the Saudi population in Riyadh (CRS) and other previously studied populations based on literature data available from the International HapMap project (http://hapmap.ncbi.nlm.nih.gov/). The results for the selected SNPs are presented in Tables 8–10. The allelic and genotypic frequencies for the P53 SNP rs1042522 differed greatly in the European (CEU; p < 0.00001) and Nigerian (YRI; p = 0.00682) populations compared with those in Saudi Arabia (Table 8). In addition, the frequencies of the various alleles of the P21 SNP rs1801270 differed greatly among the following populations: i) Utah residents with Northern and Western European ancestry (CEU) whose samples were collected in Paris, France at The Foundation Jean Dausset-CEPH (formerly known as the Center of Study of Human Polymorphisms); ii) Han Chinese residents from Beijing, China (HCB); iii) Chinese residents from Metropolitan Denver, Colorado, USA (CHD); iv) Japanese residents from Tokyo, Japan (JPT); v) Luhya residents from Webuye, Kenya (LWK); and vi) Italian residents from Tuscany, Italy (TSI). The frequencies in all of these populations differed greatly from those of the Saudis from Riyadh (p < 0.005) (Table 9). For the MDM2 SNP rs769412, there were also significant differences in the CRS population for the rs769412 genotype compared with the populations of JPT (p = 0.001857) and LWK (p = 0.001266) (Table 10).

**Table 8:** Comparison of P53 SNP frequency in different populations.

SNP ID	Populations	Samples (N)	Genotype frequency N (%)		X^2^	P value
			C	G		
rs1042522	**CEU**	120	28 (0.23)	92 (0.77)	27.54	**<0.00001**
	**HCB**	90	43 (0.48)	47 (0.52)	0.354	0.55145
	**JPT**	88	39 (0.44)	49 (0.56)	3.650	0.05604
	**YRI**	118	78 (0.66)	40 (0.34)	7.318	**0.00682**
	**CRS**	285	147 (0.52)	138 (0.48)	Refs	

**CEU:** Utah residents with Northern and Western European ancestry from the CEPH collection. **HCB:** Han Chinese in Beijing, China. **JPT:** Japanese in Tokyo, Japan. **YRI:** Yoruba in Ibadan, Nigeria. **CRS:** Saudi population residing in the Riyadh region of central Saudi Arabia.

**Table 9:** Comparison of P21 variant frequency in other populations.

SNP ID	Populations	Samples (N)	Genotype frequency N (%)		X^2^	P value
			A	C		
rs1801270	**CEU**	224	9 (0.04)	215 (0.96)	37.17	**<0.00001**
	**HCB**	86	40 (0.47)	46 (0.53)	16.62	**<0.00001**
	**JPT**	170	66 (0.39)	104 (0.61)	12.26	**0.000462**
	**YRI**	224	69 (0.31)	155 (0.69)	2.697	0.100554
	**ASW**	98	26 (0.27)	72 (0.73)	0.179	0.672645
	**CHD**	168	86 (0.51)	82 (0.49)	35.86	**<0.00001**
	**GIH**	176	32 (0.18)	144 (0.82)	1.811	0.178423
	**LWK**	180	58 (0.32)	122 (0.68)	4.218	**0.039999**
	**MEX**	98	25 (0.26)	73 (0.74)	0.139	0.709696
	**MKK**	284	74 (0.26)	210 (0.74)	0.024	0.876376
	**TSI**	176	18 (0.10)	158 (0.90)	15.03	**0.000106**
	**CRS**	274	66 (0.24)	208 (0.76)	Refs	

**CEU:** Utah residents with Northern and Western European ancestry from the CEPH collection. **HCB:** Han Chinese in Beijing, China. **JPT:** Japanese in Tokyo, Japan. **YRI:** Yoruba in Ibadan, Nigeria. **ASW:** African ancestry in Southwest USA. **CHD:** Chinese in Metropolitan Denver, Colorado. **GIH:** Gujarati Indians in Houston, Texas. **LWK**: Luhya in Webuye, Kenya. **MEX:** Mexican ancestry in Los Angeles, California. **MKK:** Maasai in Kinyawa, Kenya. **TSI:** Toscans in Italy. **CRS:** Saudi population residing in the Riyadh region of central Saudi Arabia.

**Table 10:** Genotype frequency of MDM2 polymorphism in the Saudi population compared with other populations.

SNP ID	Populations	Samples (N)	Genotype frequency N (%)		X^2^	P value
			A	G		
rs769412	**CEU**	226	210 (0.93)	16 (0.07)	0.708	0.400258
	**HCB**	86	83 (0.97)	3 (0.03)	2.075	0.149684
	**JPT**	172	170 (0.99)	2 (0.01)	9.685	**0.001857**
	**YRI**	226	201 (0.89)	25 (0.11)	1.465	0.226213
	**ASW**	98	84 (0.86)	13 (0.13)	2.477	0.115505
	**CHD**	170	163 (0.96)	7 (0.04)	2.568	0.109046
	**GIH**	174	160 (0.92)	14 (0.08)	0.035	0.851414
	**LWK**	180	148 (0.82)	32 (0.18)	10.391	**0.001266**
	**MEX**	100	92 (0.92)	8 (0.08)	0.000	1.000000
	**MKK**	286	252 (0.88)	34 (0.12)	2.526	0.111949
	**TSI**	176	164 (0.93)	12 (0.07)	0.187	0.665383
	**CRS**	282	259 (0.92)	23 (0.08)	Refs	

**CEU:** Utah residents with Northern and Western European ancestry from the CEPH collection. **HCB:** Han Chinese in Beijing, China. **JPT:** Japanese in Tokyo, Japan. **YRI:** Yoruba in Ibadan, Nigeria. **ASW:** African ancestry in Southwest USA. **CHD:** Chinese in Metropolitan Denver, Colorado. **GIH:** Gujarati Indians in Houston, Texas. **LWK**: Luhya in Webuye, Kenya. **MEX:** Mexican ancestry in Los Angeles, California. **MKK:** Maasai in Kinyawa, Kenya. **TSI:** Toscans in Italy. **CRS:** Saudi population residing in the Riyadh region of central Saudi Arabia.

## Discussion

In humans, cells have several protective pathways that can inhibit damaged DNA from duplicating itself and prevent DNA damage from occurring within self-replication pathways [58]. Genes encoding components of the P53 protein pathway are among the most widely recognized protective factors acting as tumor suppressors [56]. Nicknamed the “guardian of the genome,” the function of the TP53 pathway is essentially a protective one. Previous findings support our hypothesis that there is an association between TSU and SNP in TP53, in the context of the development of TSU‐related diseases. Although SNPs can contribute to the effectiveness of DNA repair by altering protein functions [57, 59], genetic variations caused by TSU bring about the development of several different cancer types in humans. Since a prior literature review has failed to identify any effects of TSU in generating genetic variations in components of the pathway of the TP53 gene, in the present research, we investigated the potential relationship between TSU and genetic components, especially the exonic genetic polymorphism rs1042522 in the P53 gene, the rs1801270 in the P21 gene, and the rs769412 in the MDM2 gene, which may lead to the progression of numerous diseases in humans. To investigate and identify the genetic markers that could help in determining how to decrease the risk of TSU-related diseases, we studied a sample of smokers and non-smokers from the Saudi population. A general significant association was observed in the effects of TSU on the gene polymorphisms of P53 (rs1042522) and P21 (rs1801270) in the sample of smokers in the present study. However, neither genetic nor allelic alterations were found to have occurred at SNP rs769412and MDM2, although they were found to have occurred on the P53 variant, in the Saudi smokers and non-smoking controls. The P53 polymorphism rs1042522, located in exon 4 at codon 72, involves the missense substitution C>G, which causes an amino acid transversion of proline (Pro) to arginine (Arg). In other words, amino acid transversions alter the function of P53’s protein [60]. Given the fundamental role of the P53 polymorphism rs1042522 in tumorigenesis, many researchers are attempting to explain the mechanism by which it affects the risk of developing certain cancer types. Notably, the results of the present study demonstrate that the genotype and allele frequencies of P53 rs1042522 for all clinical parameters that we tested, including age, sex, number of years smoking, daily consumption levels, and substance type, are higher in smokers than in non-smokers, thus contributing to their greater risk of developing TSU-related diseases. Similar results were found for the P53 SNP rs1042522 in other populations suffering from various malignancies [52, 61–64]. For example, previous studies found associations between rs1042522 and an increased susceptibility to developing thyroid cancer in the Indian population [61] and of developing lung adenocarcinoma in the Chinese female population [52]. The genetic variant of P21, Ser31Arg rs1801270, located at the polymorphic site codon 31, also induces changes in amino acids by mutating serine (Ser) into arginine (Arg). As an SNP located in an exonic region, it might also alter the function of P21 [65]. Several other studies have mentioned an association between the polymorphism rs1801270 and the risk of developing any of several types of malignancies, including gastric, breast, and cervical cancers. Although rs1801270 exerts several effects on a great number of cancer types [66], the results of studies on this subject remain inconclusive [67–69]. One such previous study conducted on a population of Chinese women found an association between the A allele of P21 rs1801270 and decreased cervical cancer susceptibility [68]. However, Taghavi et al. stated that in northeastern Iran, rs1801270 is not a genetic biomarker for esophageal carcinoma [44]. The results of the present study indicate that the P21 rs1801270 C allele increases the risk of developing TSU-related diseases. Surprisingly, the increase in risk was higher in younger tobacco smokers and shisha users who had been consuming tobacco products for less than seven years than the rest of the cohort. Although our test results for the polymorphism of the C allele for rs1801270 resemble those in previous reports [70–72], they also differ [73, 74] in terms of our discovery of its deleterious effects on smokers. This discrepancy might have been caused by the different demographics and populations sampled, including the ethnic groups studied, the sample sizes, and the genetic makeup (genotype). In addition, there could have been impacts of other demographic factors influencing health by causing morbidity and mortality disparities overall, such as ethnicity, social identity, economic status, environment, and culture [75] Our study found that age significantly (p < 0.05) influences the association between TSU and the C allele for P21 rs1801270. In addition, for the MDM2 gene encoding a negative regulator of P53 [76] involved in tumor growth and metastasis [77], the SNP rs769412 generates an A>G base change at codon 354. However, despite the fact that it causes binding on the Sp1 site, as a variant, it plays no part in causing amino acid substitution at rs769412. For example, in the African-American population, it showed no associations with lung cancer in smokers [78]. Moreover, in a study by Rajaraman et al. (2007), the MDM2 polymorphism rs769412 was instead found to have a protective effect against glioma [79]. Our study investigated this MDM2 variant in smokers versus non-smokers in the Saudi Arabian population for the first time. Notably, we found that it had no significant association with smoking in either of our cohorts. Therefore, our findings are similar to those of most previous studies in this area involving various populations [48, 76, 78, 80], except for the research conducted by Rajaraman et al. (2007) [79]. The results that we obtained are a useful addition to currently existing findings on the effects of TSU on an MDM2 SNP that causes the development of various malignant diseases in smokers. However, we recommend further confirmatory studies on the MDM2 SNP rs769412. It should also be noted that the number of females who participated in our study was insufficient to determine with any great statistical precision any findings regarding the polymorphisms of P21 and MDM2 in this sex. Therefore, to confirm our findings, we strongly suggest the performance of more studies on the female Saudi population through functional analysis and sampling of larger cohorts. The findings can then be compared in terms of the various demographic factors with those of other populations. There is also a need to investigate in greater detail the relationship between TSU and mutations in P53, P21, and MDM2 that lead to the development of malignant diseases. Finally, since several previous studies, including our own, have documented the harmful effects of smoking on the health of younger people and their greater risk of developing breast cancer [81, 82], we strongly recommend anti-smoking campaigns to specifically focus particularly on adolescents and young adults and help them choose a healthy lifestyle by avoiding or quitting smoking.

## Conclusions

Our results demonstrate the higher effector role of the P53 SNP rs1042522 polymorphism in all clinical parameters that increase smokers’ potential risk of developing TSU‐related diseases. We believe that more studies involving the Saudi population are warranted to discover novel diagnostic biomarkers that would assist in the early detection and diagnosis of TSU-related diseases.

## Supporting information

S1 Table Description of the selected polymorphisms. (DOCX)

## Abbreviations

TSU: tobacco substance usage
SNPs: single nucleotide polymorphisms
EDTA: ethylenediaminetetraacetic acid
CI: confidence interval
OR: odds ratio
HWE: Hardy-Weinberg equilibrium (HWE)
Pro: Proline
Arg: Arginine
Ser: Serine
Glu: Glutamic Acid.

## References

[1] Jha P., Avoidable global cancer deaths and total deaths from smoking. Nat Rev Cancer, 2009 9(9): p. 655–64. 10.1038/nrc2703 19693096

[2] Boyle P., Cancer, cigarette smoking and premature death in Europe: a review including the Recommendations of European Cancer Experts Consensus Meeting, Helsinki, October 1996. Lung Cancer, 1997 17(1): p. 1–60. 10.1016/s0169-5002(97)00648-x 9194026

[3] Jha P., et al, Tobacco Addiction, in *Disease Control Priorities in Developing Countries*, nd, et al., Editors. 2006: Washington (DC). 21250309

[4] Khuder S.A., Effect of cigarette smoking on major histological types of lung cancer: a meta-analysis. Lung Cancer, 2001 31(2–3): p. 139–48. 10.1016/s0169-5002(00)00181-1 11165392

[5] Liu X., et al, The mortality of lung cancer attributable to smoking among adults in China and the United States during 1990–2017. Cancer Commun (Lond), 2020 40(11): p. 611–619. 10.1002/cac2.12099 33029952 PMC7668493

[6] Kasajova P., et al, Active cigarette smoking and the risk of breast cancer at the level of N-acetyltransferase 2 (NAT2) gene polymorphisms. Tumour Biol, 2016 37(6): p. 7929–37. 10.1007/s13277-015-4685-3 26700672

[7] Xu Z., Xu H., and Lu Y., Genetic Liability to Smoking and Breast Cancer Risk. Clin Epidemiol, 2020 12: p. 1145–1148. 10.2147/CLEP.S270509 33116908 PMC7585802

[8] Morse D.E., et al, Smoking and drinking in relation to oral cancer and oral epithelial dysplasia. Cancer Causes Control, 2007 18(9): p. 919–29. 10.1007/s10552-007-9026-4 17647085 PMC2139900

[9] Irani S., Barati I., and Badiei M., Periodontitis and oral cancer—current concepts of the etiopathogenesis. Oncol Rev, 2020 14(1): p. 465 10.4081/oncol.2020.465 32231765 PMC7097927

[10] Blackford A., et al, Genetic mutations associated with cigarette smoking in pancreatic cancer. Cancer Res, 2009 69(8): p. 3681–8. 10.1158/0008-5472.CAN-09-0015 19351817 PMC2669837

[11] Molina-Montes E., et al, Pancreatic Cancer Risk in Relation to Lifetime Smoking Patterns, Tobacco Type, and Dose-Response Relationships. Cancer Epidemiol Biomarkers Prev, 2020 29(5): p. 1009–1018. 10.1158/1055-9965.EPI-19-1027 32051190

[12] Kytola V., et al, Mutational Landscapes of Smoking-Related Cancers in Caucasians and African Americans: Precision Oncology Perspectives at Wake Forest Baptist Comprehensive Cancer Center. Theranostics, 2017 7(11): p. 2914–2923. 10.7150/thno.20355 28824725 PMC5562225

[13] Akter S., et al, Smoking and colorectal cancer: A pooled analysis of 10 population-based cohort studies in Japan. Int J Cancer, 2020 10.1002/ijc.33248 32761607

[14] Ganguly S., Chandra A., and Chatterjee I.B., Pathobiology of cigarette smoke-induced invasive cancer of the renal pelvis and its prevention by vitamin C. Toxicol Rep, 2018 5: p. 1002–1010. 10.1016/j.toxrep.2018.10.005 30338226 PMC6186955

[15] Gansler T., et al, Prevalence of Cigarette Smoking among Patients with Different Histologic Types of Kidney Cancer. Cancer Epidemiol Biomarkers Prev, 2020 29(7): p. 1406–1412. 10.1158/1055-9965.EPI-20-0015 32357956 PMC9661925

[16] West R., Tobacco smoking: Health impact, prevalence, correlates and interventions. Psychol Health, 2017 32(8): p. 1018–1036. 10.1080/08870446.2017.1325890 28553727 PMC5490618

[17] Cerveri I., et al, The impact of cigarette smoking on asthma: a population-based international cohort study. Int Arch Allergy Immunol, 2012 158(2): p. 175–83. 10.1159/000330900 22286571 PMC3696371

[18] Harvey B.G., et al, Modification of gene expression of the small airway epithelium in response to cigarette smoking. J Mol Med (Berl), 2007 85(1): p. 39–53. 10.1007/s00109-006-0103-z 17115125

[19] Pintarelli G., et al, Cigarette smoke alters the transcriptome of non-involved lung tissue in lung adenocarcinoma patients. Sci Rep, 2019 9(1): p. 13039 10.1038/s41598-019-49648-2 31506599 PMC6736939

[20] Vink J.M., et al, Differential gene expression patterns between smokers and non-smokers: cause or consequence? Addict Biol, 2017 22(2): p. 550–560. 10.1111/adb.12322 26594007 PMC5347870

[21] Bosse Y., et al, Molecular signature of smoking in human lung tissues. Cancer Res, 2012 72(15): p. 3753–63. 10.1158/0008-5472.CAN-12-1160 22659451

[22] Landi M.T., et al, Gene expression signature of cigarette smoking and its role in lung adenocarcinoma development and survival. PLoS One, 2008 3(2): p. e1651 10.1371/journal.pone.0001651 18297132 PMC2249927

[23] Kim S.M., et al, The cigarette smoke components induced the cell proliferation and epithelial to mesenchymal transition via production of reactive oxygen species in endometrial adenocarcinoma cells. Food Chem Toxicol, 2018 121: p. 657–665. 10.1016/j.fct.2018.09.023 30236600

[24] Wahyuningsih L., et al, Tissue P16 is Associated with Smoking Status among Indonesian Nasopharyngeal Carcinoma Subjects. Asian Pac J Cancer Prev, 2019 20(7): p. 2125–2130. 10.31557/APJCP.2019.20.7.2125 31350975 PMC6745211

[25] An A.R., et al, Association between Expression of 8-OHdG and Cigarette Smoking in Non-small Cell Lung Cancer. J Pathol Transl Med, 2019 53(4): p. 217–224. 10.4132/jptm.2019.02.20 30853705 PMC6639704

[26] Iwakuma T. and Lozano G., MDM2, an introduction. Mol Cancer Res, 2003 1(14): p. 993–1000. 14707282

[27] Weng M.W., et al, Alternative splicing of MDM2 mRNA in lung carcinomas and lung cell lines. Environ Mol Mutagen, 2005 46(1): p. 1–11. 10.1002/em.20118 15714438

[28] Zwiers A., et al, Celiac disease associated SNP rs17810546 is located in a gene silencing region. Gene, 2020 726: p. 144165 10.1016/j.gene.2019.144165 31726085

[29] Ruiz-Noa Y., et al, PTPN22 1858C>T polymorphism is associated with increased CD154 expression and higher CD4+ T cells percentage in rheumatoid arthritis patients. J Clin Lab Anal, 2019 33(3): p. e22710 10.1002/jcla.22710 30402903 PMC6818609

[30] Lee K.Y., et al, Genetic association study of CSNK1E gene in bipolar disorder and circadian characteristics. Nord J Psychiatry, 2018 72(8): p. 599–604. 10.1080/08039488.2018.1509125 30445897

[31] Rajaram M., et al, Effects of genetic polymorphisms in Vitamin D metabolic pathway on Vitamin D level and asthma control in South Indian patients with bronchial asthma. Lung India, 2019 36(6): p. 483–491. 10.4103/lungindia.lungindia_23_19 31670295 PMC6852217

[32] Zhao S., Zhang W., and Nie X., Association of beta2-adrenergic receptor gene polymorphisms (rs1042713, rs1042714, rs1042711) with asthma risk: a systematic review and updated meta-analysis. BMC Pulm Med, 2019 19(1): p. 202 10.1186/s12890-019-0962-z 31699066 PMC6836544

[33] Wang S., et al, LncRNA NEAT1 polymorphisms and lung cancer susceptibility in a Chinese Northeast Han Population: A case-control study. Pathol Res Pract, 2019 215(12): p. 152723 10.1016/j.prp.2019.152723 31704150

[34] Gao J., et al, Involvement of SLC39A6 in gastric adenocarcinoma and correlation of the SLC39A6 polymorphism rs1050631 with clinical outcomes after resection. BMC Cancer, 2019 19(1): p. 1069 10.1186/s12885-019-6222-z 31703635 PMC6839152

[35] Gagno S., et al, A TGF-beta associated genetic score to define prognosis and platinum sensitivity in advanced epithelial ovarian cancer. Gynecol Oncol, 2020 156(1): p. 233–242. 10.1016/j.ygyno.2019.10.019 31711657

[36] Wang Y., et al, Impact of WNT1-inducible signaling pathway protein-1 (WISP-1) genetic polymorphisms and clinical aspects of breast cancer. Medicine (Baltimore), 2019 98(44): p. e17854 10.1097/MD.0000000000017854 31689877 PMC6946553

[37] Deng N., et al, Single nucleotide polymorphisms and cancer susceptibility. Oncotarget, 2017 8(66): p. 110635–110649. 10.18632/oncotarget.22372 29299175 PMC5746410

[38] Kruger M., et al, The impact of cigarette smoke on activity of single nucleotide polymorphisms of the vascular endothelial growth factor-promoter gene in cells of the upper aerodigestive tract. J Oral Pathol Med, 2019 48(9): p. 810–816. 10.1111/jop.12893 31166634

[39] Luetragoon T., et al, Interaction among smoking status, single nucleotide polymorphisms and markers of systemic inflammation in healthy individuals. Immunology, 2018 154(1): p. 98–103. 10.1111/imm.12864 29140561 PMC5904710

[40] Andersson B.A., et al, Cigarette smoking affects microRNAs and inflammatory biomarkers in healthy individuals and an association to single nucleotide polymorphisms is indicated. Biomarkers, 2019 24(2): p. 180–185. 10.1080/1354750X.2018.1539764 30375257

[41] Yadav P., et al, Genetic Factors Interact With Tobacco Smoke to Modify Risk for Inflammatory Bowel Disease in Humans and Mice. Gastroenterology, 2017 153(2): p. 550–565. 10.1053/j.gastro.2017.05.010 28506689 PMC5526723

[42] Mizuno S., et al, p53 Signaling Pathway Polymorphisms Associated With Emphysematous Changes in Patients With COPD. Chest, 2017 152(1): p. 58–69. 10.1016/j.chest.2017.03.012 28315337

[43] Chandirasekar R., et al, Assessment of genotoxic and molecular mechanisms of cancer risk in smoking and smokeless tobacco users. Mutat Res Genet Toxicol Environ Mutagen, 2014 767: p. 21–7. 10.1016/j.mrgentox.2014.04.007 24769293

[44] Taghavi N., et al, P21(waf1/cip1) gene polymorphisms and possible interaction with cigarette smoking in esophageal squamous cell carcinoma in northeastern Iran: a preliminary study. Arch Iran Med, 2010 13(3): p. 235–42. 20433229

[45] Merugu S., et al, Detection of Circulating and Disseminated Neuroblastoma Cells Using the ImageStream Flow Cytometer for Use as Predictive and Pharmacodynamic Biomarkers. Clin Cancer Res, 2020. 26(1): p. 122–134. 10.1158/1078-0432.CCR-19-0656 31767563

[46] Abdel Hamid T.M., et al, Clinical impact of SNP of P53 genes pathway on the adult AML patients. Hematology, 2015 20(6): p. 328–35. 10.1179/1607845414Y.0000000200 25232917

[47] Dumont P., et al, The codon 72 polymorphic variants of p53 have markedly different apoptotic potential. Nat Genet, 2003 33(3): p. 357–65. 10.1038/ng1093 12567188

[48] Jiao Y., et al, A Functional Polymorphism (rs937283) in the MDM2 Promoter Region is Associated with Poor Prognosis of Retinoblastoma in Chinese Han Population. Sci Rep, 2016 6: p. 31240 10.1038/srep31240 27506496 PMC4979029

[49] Wang H. and Ma K., Association between MDM2 rs769412 and rs937283 polymorphisms with alcohol drinking and laryngeal carcinoma risk. Int J Clin Exp Pathol, 2015 8(6): p. 7436–40. 26261649 PMC4525983

[50] Salimi S., et al, The effects of p21 gene C98A polymorphism on development of uterine leiomyoma in southeast Iranian women. Tumour Biol, 2016 37(9): p. 12497–12502. 10.1007/s13277-016-5078-y 27337955

[51] Harati-Sadegh M., et al, Analysis of polymorphisms, promoter methylation, and mRNA expression profile of maternal and placental P53 and P21 genes in preeclamptic and normotensive pregnant women. J Biomed Sci, 2019 26(1): p. 92 10.1186/s12929-019-0586-x 31703578 PMC6842146

[52] Ren Y.W., et al, P53 Arg72Pro and MDM2 SNP309 polymorphisms cooperate to increase lung adenocarcinoma risk in Chinese female non-smokers: a case control study. Asian Pac J Cancer Prev, 2013 14(9): p. 5415–20. 10.7314/apjcp.2013.14.9.5415 24175836

[53] Hancox R.J., et al, Accelerated decline in lung function in cigarette smokers is associated with TP53/MDM2 polymorphisms. Hum Genet, 2009 126(4): p. 559–65. 10.1007/s00439-009-0704-z 19521721 PMC3740961

[54] Kelley L.A., et al, The Phyre2 web portal for protein modeling, prediction and analysis. Nat Protoc, 2015 10(6): p. 845–58. 10.1038/nprot.2015.053 25950237 PMC5298202

[55] Almutairi M., et al, Effect of the thymine-DNA glycosylase rs4135050 variant on Saudi smoker population. Mol Genet Genomic Med, 2019 7(4): p. e00590 10.1002/mgg3.590 30779328 PMC6465727

[56] Brazda V., et al, DNA and RNA quadruplex-binding proteins. Int J Mol Sci, 2014 15(10): p. 17493–517. 10.3390/ijms151017493 25268620 PMC4227175

[57] de Boer J.G., Polymorphisms in DNA repair and environmental interactions. Mutat Res, 2002 509(1–2): p. 201–10. 10.1016/s0027-5107(02)00217-8 12427539

[58] Pages V. and Fuchs R.P., How DNA lesions are turned into mutations within cells? Oncogene, 2002 21(58): p. 8957–66. 10.1038/sj.onc.1206006 12483512

[59] Xi T., Jones I.M., and Mohrenweiser H.W., Many amino acid substitution variants identified in DNA repair genes during human population screenings are predicted to impact protein function. Genomics, 2004 83(6): p. 970–9. 10.1016/j.ygeno.2003.12.016 15177551

[60] Grochola L.F., et al, Single-nucleotide polymorphisms in the p53 signaling pathway. Cold Spring Harb Perspect Biol, 2010 2(5): p. a001032 10.1101/cshperspect.a001032 20452958 PMC2857176

[61] Khan M.S., et al, Significant association of TP53 Arg72Pro polymorphism in susceptibility to differentiated thyroid cancer. Cancer Biomark, 2015 15(4): p. 459–65. 10.3233/CBM-150485 25835179 PMC12965092

[62] Kumari A., et al, Association of p53 codon 72 polymorphism and survival of North Indian lung cancer patients treated with platinum-based chemotherapy. Mol Biol Rep, 2016 43(12): p. 1383–1394. 10.1007/s11033-016-4072-1 27614750

[63] Li Y., et al, TP53 genetic polymorphisms, interactions with lifestyle factors and lung cancer risk: a case control study in a Chinese population. BMC Cancer, 2013 13: p. 607 10.1186/1471-2407-13-607 24369748 PMC3877976

[64] Lin H.Y., et al, Polymorphisms of TP53 are markers of bladder cancer vulnerability and prognosis. Urol Oncol, 2013 31(7): p. 1231–41. 10.1016/j.urolonc.2011.11.031 22178231

[65] Chedid M., et al, A single nucleotide substitution at codon 31 (Ser/Arg) defines a polymorphism in a highly conserved region of the p53-inducible gene WAF1/CIP1. Oncogene, 1994 9(10): p. 3021–4. 8084608

[66] Xiao F., et al, Association between the ERCC2 Asp312Asn polymorphism and risk of cancer. Oncotarget, 2017 8(29): p. 48488–48506. 10.18632/oncotarget.17290 28489582 PMC5564664

[67] Li Y., et al, P21 Ser31Arg polymorphism and cervical cancer risk: a meta-analysis. Int J Gynecol Cancer, 2011 21(3): p. 445–51. 10.1097/IGC.0b013e31820da58b 21430453

[68] Wang N., et al, Association of p21 SNPs and risk of cervical cancer among Chinese women. BMC Cancer, 2012 12: p. 589 10.1186/1471-2407-12-589 23231583 PMC3527144

[69] Qiu L.X., et al, The p21 Ser31Arg polymorphism and breast cancer risk: a meta-analysis involving 51,236 subjects. Breast Cancer Res Treat, 2010 124(2): p. 475–9. 10.1007/s10549-010-0858-3 20349127

[70] Li G., et al, Genetic polymorphisms of p21 are associated with risk of squamous cell carcinoma of the head and neck. Carcinogenesis, 2005 26(9): p. 1596–602. 10.1093/carcin/bgi105 15878916

[71] Powell B.L., et al, Associations between common polymorphisms in TP53 and p21WAF1/Cip1 and phenotypic features of breast cancer. Carcinogenesis, 2002 23(2): p. 311–5. 10.1093/carcin/23.2.311 11872638

[72] Wu M.T., et al, Association between p21 codon 31 polymorphism and esophageal cancer risk in a Taiwanese population. Cancer Lett, 2003 201(2): p. 175–80. 10.1016/s0304-3835(03)00469-5 14607331

[73] Polakova V., et al, Genotype and haplotype analysis of cell cycle genes in sporadic colorectal cancer in the Czech Republic. Hum Mutat, 2009 30(4): p. 661–8. 10.1002/humu.20931 19224585

[74] Shih C.M., et al, Lack of evidence of association of p21WAF1/CIP1 polymorphism with lung cancer susceptibility and prognosis in Taiwan. Jpn J Cancer Res, 2000 91(1): p. 9–15. 10.1111/j.1349-7006.2000.tb00854.x 10744039 PMC5926229

[75] Birgander R., et al, The codon 31 polymorphism of the p53-inducible gene p21 shows distinct differences between major ethnic groups. Hum Hered, 1996 46(3): p. 148–54. 10.1159/000154344 8860009

[76] Reza H.A., et al, MDM2 SNP 285 is Associated with Reduced Lung Cancer Risk in Bangladeshi Population. Mymensingh Med J, 2020 29(1): p. 108–114. 31915345

[77] Qiu Y.L., et al, Genetic polymorphisms, messenger RNA expression of p53, p21, and CCND1, and possible links with chromosomal aberrations in Chinese vinyl chloride-exposed workers. Cancer Epidemiol Biomarkers Prev, 2008 17(10): p. 2578–84. 10.1158/1055-9965.EPI-07-2925 18842998

[78] Pine S.R., et al, MDM2 SNP309 and SNP354 are not associated with lung cancer risk. Cancer Epidemiol Biomarkers Prev, 2006 15(8): p. 1559–61. 10.1158/1055-9965.EPI-06-0217 16896050

[79] Rajaraman P., et al, Polymorphisms in apoptosis and cell cycle control genes and risk of brain tumors in adults. Cancer Epidemiol Biomarkers Prev, 2007 16(8): p. 1655–61. 10.1158/1055-9965.EPI-07-0314 17684142

[80] Boersma B.J., et al, Association of breast cancer outcome with status of p53 and MDM2 SNP309. J Natl Cancer Inst, 2006 98(13): p. 911–9. 10.1093/jnci/djj245 16818855

[81] Verde Z., et al, Effect of Genetic Polymorphisms and Long-Term Tobacco Exposure on the Risk of Breast Cancer. Int J Mol Sci, 2016 17(10). 10.3390/ijms17101726 27754415 PMC5085757

[82] Perez-Rubio G., et al, Genetic polymorphisms in CYP2A6 are associated with a risk of cigarette smoking and predispose to smoking at younger ages. Gene, 2017 628: p. 205–210. 10.1016/j.gene.2017.07.051 28734893

