# Supplementary material for: Association between tobacco substance usage and a missense mutation in the tumor suppressor gene P53 in the Saudi Arabian population
Source: PLoS One. 2021 Jan 22;16(1):e0245133. doi: 10.1371/journal.pone.0245133 (PMC7822264; doi:10.1371/journal.pone.0245133)
Supplement: S1 Table — (DOCX) [file pone.0245133.s001.docx]

S1 Table. Description of the selected polymorphisms

| **Gene** | **SNP ID** | **SNP location** | **Variation type** | **Alleles change** | **Amino acids change** |
| --- | --- | --- | --- | --- | --- |
| *TP53* | rs1042522 | NC_000017.11:g.7676154 | Exon | C/G | P72R |
| *P21* | rs1801270 | NC_000006.12:g.36684194 | Exon | A/C | R31S |
| *MDM2* | rs769412 | NC_000012.12:g.68839435 | Exon | A/G | E354E |

Abbreviations: SNP: Single Nucleotide Polymorphism; P: Proline; R: Arginine; S: Serine; E: Glutamic Acid.
